# Maternal warmth is associated with network segregation across late childhood: A longitudinal neuroimaging study

**DOI:** 10.3389/fpsyg.2022.917189

**Published:** 2022-09-13

**Authors:** Sally Richmond, Richard Beare, Katherine A. Johnson, Katherine Bray, Elena Pozzi, Nicholas B. Allen, Marc L. Seal, Sarah Whittle

**Affiliations:** ^1^Melbourne Neuropsychiatry Centre, Department of Psychiatry, The University of Melbourne and Melbourne Health, Parkville, VIC, Australia; ^2^Turner Institute for Brain and Mental Health, School of Psychological Sciences, Monash University, Melbourne, VIC, Australia; ^3^Developmental Imaging, Murdoch Children’s Research Institute, Parkville, VIC, Australia; ^4^Melbourne School of Psychological Sciences, The University of Melbourne, Parkville, VIC, Australia; ^5^Department of Psychology, University of Oregon, Eugene, OR, United States; ^6^Department of Pediatrics, The University of Melbourne, Parkville, VIC, Australia

**Keywords:** cortical thickness, longitudinal, graph theory, parenting, magnetic resonance imaging

## Abstract

The negative impact of adverse experiences in childhood on neurodevelopment is well documented. Less attention however has been given to the impact of variations in “normative” parenting behaviors. The influence of these parenting behaviors is likely to be marked during periods of rapid brain reorganization, such as late childhood. The aim of the current study was to investigate associations between normative parenting behaviors and the development of structural brain networks across late childhood. Data were collected from a longitudinal sample of 114 mother-child dyads (54% female children, *M* age 8.41 years, SD = 0.32 years), recruited from low socioeconomic areas of Melbourne, Australia. At the first assessment parenting behaviors were coded from two lab-based interaction tasks and structural magnetic resonance imaging (MRI) scans of the children were performed. At the second assessment, approximately 18 months later (*M* age 9.97 years, SD = 0.37 years) MRI scans were repeated. Cortical thickness (CT) was extracted from T1-weighted images using FreeSurfer. Structural covariance (SC) networks were constructed from partial correlations of CT estimates between brain regions and estimates of network efficiency and modularity were obtained for each time point. The change in these network measures, from Time 1 to Time 2, was also calculated. At Time 2, less positive maternal affective behavior was associated with higher modularity (more segregated networks), while negative maternal affective behavior was not related. No support was found for an association between local or global efficacy and maternal affective behaviors at Time 2. Similarly, no support was demonstrated for associations between maternal affective behaviors and change in network efficiency and modularity, from Time 1 to Time 2. These results indicate that normative variations in parenting may influence the development of structural brain networks in late childhood and extend current knowledge about environmental influences on structural connectivity in a developmental context.

## Introduction

The profound effects of extreme parenting behaviors (e.g., involving child maltreatment and neglect) on the developing brain have been well documented (Teicher et al., 2016). Less attention has been directed toward “normative” variations in parenting behaviors which may be less severe but occur more commonly and therefore have the potential to impact a greater proportion of children (Morris et al., 2017; Farber et al., 2020; Bhanot et al., 2021). There is increasing evidence of a link between “normative” parenting behaviors and brain structure across childhood and adolescence (Belsky and de Haan, 2011; Kok et al., 2015; Cortes Hidalgo et al., 2021; Whittle et al., 2022). Findings suggest that positive (sensitive, warm, and supportive) and negative (intrusive, aggressive, and hostile) components of parenting may be differentially associated with the structure of cortical and sub-cortical regions, however results have been inconsistent. Positive parenting behaviors have been found to predict more mature growth patterns, including larger hippocampal volumes (Luby et al., 2016) and increased cortical thinning (Whittle et al., 2014). More recent studies, however, have not supported associations between positive parenting behaviors and structural brain development (Whittle et al., 2022). Negative parenting behaviors have been linked to less mature growth patterns, i.e., reduced cortical thickness (CT) and thinning across childhood and adolescence (Whittle et al., 2016, 2022; Chad-Friedman et al., 2021). Inconsistencies are also evident with negative parenting behaviors associated with smaller (Cortes Hidalgo et al., 2021) and larger (Lee et al., 2019) amygdala volumes.

Parenting behaviors are likely to be salient during late childhood, a period that begins from approximately 8 years of age. Late childhood represents an important period where unique neurodevelopmental patterns are associated with the development of internalizing and externalizing symptoms (Papachristou and Flouri, 2019; Whittle et al., 2020). Late childhood is also a time when parents are likely to be more prominent, occurring before the transition to adolescence when the influence of peers increases (Lamblin et al., 2017). As a period of intensive structural brain change, including gray and white matter organization (Petanjek et al., 2011; Vértes and Bullmore, 2015; Walhovd et al., 2017), it is likely that parenting behaviors may shape neurodevelopment during late childhood.

Additional insight into the relationship between parenting behaviors and brain structure may be garnered by applying a whole brain network approach as brain regions do not function in isolation. Functional magnetic resonance imaging (fMRI) studies indicate associations between parenting behaviors and the functional connectivity of networks during emotion processing and resting state in children. Within community samples of children, for example, associations have been demonstrated between maternal behaviors and emotion and reward processing circuitry (Pozzi et al., 2019; Kopala-Sibley et al., 2020). Similarly, associations have been found between maternal aversive behavior and resting state connectivity in the amygdala (Callaghan et al., 2017), and between inter-parental conflict and default mode network (DMN) connectivity (Graham et al., 2015). How parenting behaviors influence functional networks is unclear, however the results indicate large-scale networks are sensitive to variations in parenting behaviors.

Structural brain networks can be classified as derived from white matter (e.g., diffusion tensor imaging) or gray matter. Our focus is on gray matter networks, specifically networks based on the structural covariance (SC) of CT. SC networks, i.e., correlations between the structural properties of brain regions, may reflect synchronized changes due to shared trophic influences and other factors, including environmental (Alexander-Bloch et al., 2013a,b). Exposure to maltreatment in childhood has been associated with altered centrality (“importance” of the network nodes; Teicher et al., 2014; Sun et al., 2018a,b), reduced network clustering (grouping of the nodes within a network; Latora and Marchiori, 2001), and reduced modularity (the strength of the division into groups; Nikolova et al., 2018) in SC networks.

Structural covariance networks can be described using many metrics; here we focus on network efficiency and modularity because they (a) are fundamental network properties and are biologically relevant, (b) have been linked to family environments, and (c) demonstrate developmental changes. During late childhood (from approximately 8–12 years of age): local efficiency and modularity decrease relative to younger and older children; global efficiency increases relative to younger and older children (Khundrakpam et al., 2013; Nie et al., 2013).

Local network efficiency quantifies communication between a node (e.g., cortical gray matter region) and its immediate neighbor’s (Latora and Marchiori, 2001; Rubinov and Sporns, 2010). Local network efficiency reflects localized processing and is classified as a measure of network segregation (Rubinov and Sporns, 2010). In contrast, global network efficiency quantifies how the network sends and combines information from distributed nodes (parallel information transfer) and is considered a measure of network integration (Rubinov and Sporns, 2010; Fan et al., 2011). Modularity quantifies the degree to which clusters of nodes are densely connected to each other and sparsely connected to the rest of the network (Sporns and Betzel, 2016). Modules are theorized to allow the brain to communicate efficiently by enabling functional specialization and therefore, modularity is classified as a measure of network segregation (Sporns and Betzel, 2016).

In a cross-sectional study, our group demonstrated an association between higher levels of negative affective maternal behaviors and decreased local network efficiency and modularity in children (Richmond et al., 2019, 2021). Higher levels of positive affective maternal behaviors were associated with increased local network efficiency. No associations were found between maternal behaviors and global network efficiency or between positive affective maternal behaviors and modularity. We concluded that parenting behaviors may be related to the segregation of brain networks (as measured by local network efficiency and modularity) and thus, the ability of brain networks to process information locally. Given that local efficiency and modularity are at their lowest during late childhood (Khundrakpam et al., 2013), we speculated that the association between the segregation metrics and less optimal parenting may reflect an accelerated pattern of brain maturation. Given the cross-sectional nature of these findings and the relatively small literature base, however, interpretations about brain maturation are speculative.

A small number of studies have explored SC over time (i.e., longitudinally) in children and adolescents (Alexander-Bloch et al., 2013b; Geng et al., 2017). There are a number of ways to investigate the development of SC over time that are equally informative, including the sliding window approach (Vasa et al., 2017; Vijayakumar et al., 2021) and maturational covariance (Alexander-Bloch et al., 2013b). Maturational covariance is the correlation between each brain region’s rate of change (e.g., thickness/year) across subjects (Alexander-Bloch et al., 2013b). Maturational covariance may clarify the phenomenon of SC if it can be shown that there are coordinated changes in brain structure during development. Structural (cross-sectional) and maturational (longitudinal) networks have been found to share similar global and nodal topological properties (sample of 9–22 year olds; Alexander-Bloch et al., 2013b). Seed based investigations based on the default, dorsal attention, primary visual, and motor networks have also demonstrated pairings in rates of change of between nodes (e.g., cortical gray matter regions) which are similar to corresponding functional networks (Geng et al., 2017).

The aim of the current study was to investigate the longitudinal relationship between parenting behaviors and the properties of structural brain networks, specifically efficiency and modularity, as measured by SC of CT, from 8 to 10 years of age. We expected to see similar network patterns at age 10 to those that we found at age 8, i.e., higher levels of negative parenting behaviors associated with decreased local efficiency and modularity; higher levels of positive maternal behaviors associated with increased local efficiency; and no associations between parenting behaviors and global network efficiency (Richmond et al., 2019, 2021). We hypothesized that non-optimal parenting behaviors, i.e., more negative and less positive behaviors, would be associated with lower local efficiency and lower modularity at age 10. We also expected, given (a) local efficiency and modularity, derived from SC networks, appear to be on a downward trajectory during late childhood (Khundrakpam et al., 2013) and (b) non-optimal parenting behaviors in late childhood are associated with decreased local efficiency and modularity, that over time non-optimal parenting may accelerate changes in these network measures (Richmond et al., 2019, 2021). We also hypothesized that non-optimal parenting behaviors would be associated with larger changes in local efficiency and modularity from age 8 to 10 (reflecting accelerated brain development), although this was a tentative hypothesis given other work showing poor parenting practices to be associated with delayed brain development (e.g., Whittle et al., 2016). We hypothesized that non-optimal parenting behaviors would not be associated with global efficiency.

## Materials and methods

### Participants and recruitment

The research was approved by The University of Melbourne Human Research Ethics Office (HREC #1339904) and written informed consent was obtained from each child and a parent/guardian. The methods and measures for the current study are provided below and the complete details for Families and Childhood Transitions Study (FACTS) be sourced in the study protocol (Simmons et al., 2017). Child-mother dyads were recruited from low socioeconomic areas of Melbourne, Australia into a longitudinal study, the FACTS. Neighborhood social disadvantage and parenting behaviors have been associated with brain development (Whittle et al., 2017). To avoid recruiting a sample biased for high socioeconomic advantage and potentially low variation in parenting behaviors, participant recruitment focused on suburbs of Melbourne that scored within the lowest tertile on the Socioeconomic Indexes for Areas scale of advantage and disadvantage (Australian Bureau of Statistics, 2013). This approach led to a sample of families with a wide range of socioeconomic status, as demonstrated by the sample’s Index of Relative Socio-Economic Advantage and Disadvantage score ranging from 1 to 94 (*M* = 41.81, SD = 24.50, *N* = 106).

Typically developing children, aged 8 years, and their mothers were invited to participate in the study. Families who expressed interest in participating were contacted for a brief telephone interview to assess the exclusion criteria, which included significant motor or sensory impairments, and criteria related to having a magnetic resonance imaging (MRI) scan.

Our previous cross-sectional work (Richmond et al., 2019, 2021) was based on 145 child-mother dyads at baseline. The current longitudinal study included 114 mother-child dyads who had family interaction and MRI data at baseline (Time 1) and MRI data at the second assessment (Time 2). See Table 1 for participant characteristics.

**TABLE 1:** Demographic and clinical participant information (*n* = 114).

Characteristic	Time 1 (*n* = 145)^a^	Time 1 (*n* = 114)	Group difference *p*	Time 2 (*n* = 114)
Child age, *M* (SD), [range] years	8.42 (0.33) [7.82–9.13]	8.42 (0.32) [7.82–9.05]	0.88	9.97 (0.37) [9.41–11.39]
Females, No. (%)	73 (52.8)	61 (53.5)	0.30	61 (53.5)
CDI-2, *M* (SD)^b^	8.32 (6.07) T-Score 55, “Average or Lower”	8.21 (6.02)	0.84	–
SCAS, *M* (SD)^c^	26.27 (13.07) T-Score 52, “Normal”	26.14 (13.28)	0.81	–
LITE, *M* (SD)^d^	3.82 (2.33)	3.79 (2.32)	0.94	–
Child ethnicity			0.08	
Caucasian, No. (%)	102 (71.03)	99 (86.8)		–
Other, No. (%)	30 (20.70)	15 (13.2)		–
Maternal age, *M* (SD), [range] years	40.25 (5.50) [28.19–52.63]	40.71 (4.94) [28.19–51.39]	0.48	–
Maternal Occupational Status, *M* (SD)^d^	62.38 (19.94)	61.53 (20.45)	0.74	–

^a^Participants with family interaction and MRI data. ^b^Imputed data, The Children’s Depression Inventory 2, maximum T-Score for boys and girls 7–12 years (Kovacs, 2011). ^c^Imputed data, The Spence Children’s Anxiety Scale maximum T-Score for boy and girls aged 8–11 years (Spence, 1998). ^d^Lifetime Incidence Traumatic Events, n = 143 (Greenwald and Rubin, 1999). ^d^Maternal occupational status from the Socioeconomic Index 2006 (AUSEI06), n = 138 (McMillan et al., 2009).

### Procedure

At Time 1, children and their mothers completed an assessment including two lab-based interaction tasks and questionnaires comprising questions about demographics, health, and developmental information. MRI scans of the children were also completed. At Time 2, approximately 18 months later MRI scans were repeated.

#### Questionnaire measures

##### The children’s depression inventory 2

The children’s depression inventory 2 (CDI-2) (Kovacs, 2011) is a 28-item child self-report measure of depressive symptoms in children and adolescents aged 7–17 years. Participants were asked to respond about symptoms experienced in the previous 2 weeks. The CDI-2 Total score and two scale scores (Emotional Problems and Functional Problems) were examined in the current study. The CDI-2 has normative data in the relevant age range and there is evidence for reliability and validity across community and clinical populations (Kovacs, 2011).

##### The spence children’s anxiety scale

The spence children’s anxiety scale (SCAS) is a 44-item child self-report measure of anxiety symptoms for children aged from 8 to 15 years (Spence, 1998). While the SCAS yields a Total score and six sub-scale scores (Obsessive-compulsive Problems, Separation Anxiety, Social Phobia, Panic/Agoraphobia, Generalized Anxiety Symptoms, and Concerns of Physical injury), only the Total score was examined in this study. There is evidence that the SCAS is a reliable and valid measure across diverse childhood populations (Essau et al., 2002; Holly et al., 2014).

##### The lifetime incidence of traumatic events

The lifetime incidence of traumatic events (LITE) (Greenwald and Rubin, 1999) is a 16-item parent-report instrument which assesses loss or trauma (e.g., fire, car accident, and hospitalization or death of friend/family member) experienced by the child. The LITE has good reliability and adequate validity (Greenwald and Rubin, 1999) but does not have a standardized scoring system. The LITE was scored by summing the number of items endorsed. The University of Melbourne Ethics Committee requested that two items on sexual abuse were removed. They were replaced with items covering mother-child separations and domestic relocation.

##### Demographics

Maternal occupational status: the Australian Socioeconomic Index 2006 (AUSEI06) is a continuous measure of occupational status, based upon 2006 Australian Census data, and ranges from 0, low status, to 100, high status (McMillan et al., 2009).

Neighborhood socio-economic status: the Index of Relative Socio-Economic Advantage and Disadvantage from the Socio-Economic Indexes for Areas is a continuous measure based on 2011 Australian census data (Australian Bureau of Statistics, 2013). Scores range from 0 to 100, where a lower score indicates an area with relatively greater disadvantage.

#### Family interaction assessment and measures

Two 15-min laboratory-based interaction tasks were completed by the mother-child dyads: (1) an event-planning interaction (EPI); and (2) a problem-solving interaction (PSI) (Gilboa and Revelle, 1994). In the EPI, dyads were asked to plan two or three activities together chosen from the Pleasant Event Schedule, such as a birthday party or holiday (MacPhillamy and Lewinsohn, 1982). In the PSI, the dyads were asked to discuss and try to resolve areas of conflict chosen from the Issue Checklist (Prinz et al., 1979). The EPI and PSI tasks were intended to differentially elicit positive and negative behaviors, respectively. Both interaction tasks were video recorded for subsequent independent coding by two extensively trained graduate students using a modified version of The Family Interaction Macro-coding system (FIMS) (Holmbeck et al., 2007).

An exploratory principal components analysis (PCA) was conducted on the FIMS mother-child data to identify empirically derived components of maternal parenting behavior (described in detail elsewhere; Richmond et al., 2018). The PCA included coded interactions for 155 mother-child dyads (10 of whom did not have MRI data). The four following components were identified: (1) *Negativity EPI*–negative maternal behaviors during the EPI, such as frequency and intensity of aggressive affect; (2) *Warmth*–codes related to positive affect, such as frequency and intensity of humor and laughter; (3) *Negativity PSI*–negative maternal behavior during the PSI; and (4) *Communication*–codes related to listening, providing explanations, and clarity of thought. For each parenting component, participant scores were estimated (Harman, 1976) and divided into two groups: low- and high- based on median split. In our earlier cross-sectional work, we divided participants into three group, low-, moderate-, and high, however due to the smaller sample size and our aim to maintain a minimum group size around 50, only two groups were possible in the current study (Richmond et al., 2019, 2021). Groups were required because SC networks are constructed from correlations (e.g., correlations of CT between brain regions) and represent a group of participants and not the network of an individual.

#### Magnetic resonance imaging acquisition and processing

To minimize the likelihood of movement artifacts, and participant anxiety a mock scanner was used to simulate the real MRI experience. Neuroimaging data were acquired on a 3T Siemens TIM Trio scanner at the Murdoch Children’s Research Institute, Melbourne. Participants were positioned supine with a 32-channel head coil. The *T*_1_-weighted image acquisition sequence was 5:19 min in duration (MPRAGE: repetition time = 2,530 ms; multiple echo times = 1.74; 3.6; 5.5; 7.3 ms; flip angle = 7°, field of view = 256 mm × 256 mm) and produced 176 contiguous 1.0 mm thick slices (voxel dimensions = 1.0 mm^3^). Motion artifacts were monitored at the time of acquisition and the sequence was repeated where possible.

#### Structural image processing

Cortical thickness, derived from the *T*_1_-weighted images, was modeled using FreeSurfer (Version 5.3; Fischl, 2012). FreeSurfer processing steps are described in detail elsewhere (Dale et al., 1999; Fischl et al., 1999). All *T*_1_-weighted images were visually inspected (all image slices for each participant) and manual edits were made where cortical surfaces were under- or over-estimated on four or more image slices. Manual edits were made to approximately one third of all scans (Time 1 and Time 2) was one scan was excluded due to excessive motion. Outlier detection, based on z-scores of CT, was applied pre- and post-manual edits to assess image quality.

#### Longitudinal stream in FreeSurfer

To address issues such as geometric distortion and voxel dimension drift, which can compromise longitudinal data, images were processed through the longitudinal stream of FreeSurfer 5.3 (Reuter et al., 2012). This stream involves the creation of a within-subject unbiased template space and average image across time points using robust, inverse consistent registration. This template is used in segmentation processes for each time point, and provides common information about anatomical structures, significantly increasing reliability and statistical power (Reuter and Fischl, 2011).

#### Missing data

There was no missing family interaction data. For the CDI-2 and the SCAS, at least 18% of child participants had missing data on one or more items. Multiple imputation at the item level was used to predict missing data. Five imputed data sets were generated, and pooled results were reported (Enders, 2010). One participant did not complete any items on the SCAS and was removed. Two participants did not complete any items on the LITE and were removed. Missing items on the LITE were assumed as not endorsed and were not imputed. Similarly, missing maternal occupational data (*n* = 7) was not imputed.

#### Structural covariance network definition

The approach to define the SC network has been defined previously (Richmond et al., 2019, 2021). The brief details are as follows, network nodes represented the FreeSurfer parcelation of the cortical gray matter regions as per the Destrieux atlas, i.e., 74 regions per hemisphere (Destrieux et al., 2010). Network edges represented the *partial* correlations of average CT between pairs of nodes (He et al., 2008; Teicher et al., 2014). Network edges (non-zero partial correlations) were selected by applying a Lasso (least absolute shrinkage and selection operator) sparse estimation technique (Tibshirani, 1996). Sparse inverse covariance methods provide variable (edge) selection by explicitly setting some coefficients to zero (Peng et al., 2009). The use of sparse inverse covariance estimates in this way has been previously demonstrated for brain networks in clinical populations to identify statistically significant group differences (Lefort-Besnard et al., 2018). All networks were analyzed as binary and undirected (i.e., edges had no orientation; Sporns, 2012).

#### Structural covariance network analysis

##### Parenting component characteristics

The influence of confounding variables on the associations between parenting behaviors and brain networks, was investigated by examining between-group differences (ANOVA) for the low- and high- groups for each of the four parenting components. The following variables were considered: child age, sex, depression symptoms, anxiety symptoms, incidence of traumatic events, and maternal occupational status. We applied an FDR (5%) to adjust for multiple comparisons (i.e., 4 parenting groups × 6 variables, 24 comparisons in total).

##### Network parameters

Brief definitions for local network efficiency, global network efficiency and modularity are provided as the definitions for these graph metrics are defined elsewhere (e.g., Fornito et al., 2016). Local efficiency, a measure of integration, was defined as the efficiency of information transfer within each subgraph or neighborhood (e.g., between a node and its first neighbors; Fornito et al., 2016). The mean local efficiency of the network was defined as the average local efficiency of all nodes. Global efficiency was defined as the average inverse of the minimum number of edges that must be traversed to go from one node to another (i.e., the characteristic path length; Latora and Marchiori, 2001; Bullmore and Sporns, 2009).

The participation coefficient was selected to define modularity and quantifies the distribution of a node’s edges across communities or modules (Guimerà and Amaral, 2005). Strong connections to many modules are indicated by a high participation coefficient, and conversely strong connections to few modules by a low participation coefficient. Subsequently, networks with high participation coefficients demonstrate less segregation between modules than networks with low participation coefficients (Baum et al., 2017).

##### Network parameter differences between parenting groups

A non-parametric permutation test procedure was applied to assess differences between network parameters (mean local efficiency, global efficiency, and modularity) between the two parenting Groups (low- and high-) for each of the four components (Negativity EPI, Warmth, Positivity PSI, Communication; Bullmore et al., 1999; He et al., 2008). All comparisons used the whole group regularization parameter. For each parenting component, the networks properties were calculated and compared (e.g., the difference in modularity for the low-Warmth and high-Warmth Groups). Participants were then randomly allocated to one of two groups and networks were constructed per the sparse partial correlation estimation procedure detailed previously. Then, for each parenting component the network properties were again calculated and compared (e.g., the difference in modularity between two random groups). The random allocation procedure was repeated 5,000 times for the three network parameters. The 95 percentile points for each distribution were used as the critical values for a two-tailed test of the null hypothesis (i.e., differences between the low- and high- Groups might occur by chance) with a probability of type 1 error of 0.05.

## Results

### Mother and child characteristics

A total of 114 mother-child dyads were included in the analysis. At Time 1, children were aged between 7 years 9 months and 9 years 0 months, and mothers between 28 and 51 years. Child reported depressive and anxiety symptoms were in the “average” range (T-score < 55, Table 1). Differences in child characteristics between all Time 1 participants (*n* = 145) and those that were analyzed, i.e., those with family interaction and MRI data, were checked using *t*-tests or Chi-squared tests. No significant group differences were found between child age, child sex, child reported depressive symptoms, child reported anxiety symptoms, parent reported incidence of lifetime traumatic events for the child, child ethnicity, maternal occupational status and maternal age (Table 1).

A logistic regression was performed to assess for any differences between the Time 2 participants and those that withdrew. The model contained five independent variables, child reported depressive symptoms, child reported anxiety symptoms, parent reported incidence of lifetime traumatic events for the child, maternal occupational status and maternal age. The full model, containing all predictors, was not statistically significant [χ^2^ (5, *N* = 127), *p* > 0.05] indicating that the model was not able to distinguish between participants at Time 1 compared to Time 2.

### Parenting group characteristics

Descriptive statistics for the low- and high- groups of each parenting component are listed in Table 2. For each parenting component (Negativity PSI, Warmth, Negativity EPI, and Communication) no significant between-group differences were found for child reported depressive symptoms, child reported anxiety symptoms, age, sex, incidence of lifetime traumatic events for the child, maternal occupational status or maternal age (FDR 5%). As a result, there was no further consideration of these variables in the analysis and partial correlations of CT were used to generate the networks.

**TABLE 2:** Group information from family interaction macro-coding system principal components analysis (*n* = 114).

	Whole group		Low-				High-			
			
Parenting Component	Skew	Kurtosis	*N*	Min	Max	Mean (SD)	*N*	Min	Max	Mean (SD)
Negativity EPI	1.98	3.86	57	−1.05	−0.36	−0.55 (0.14)	57	−0.36	4.45	0.63 (1.08)
Warmth	0.23	1.22	57	−3.49	−0.10	−0.81 (0.60)	57	−0.06	3.10	0.74 (0.72)
Negativity PSI	0.89	0.31	57	−1.37	−0.19	−0.76 (0.29)	57	−0.17	3.50	0.85 (0.79)
Communication	−1.80	4.80	57	−4.09	−0.31	−0.52 (0.96)	57	0.32	2.01	0.76 (0.32)

### Resolution parameter

The resolution parameter was calculated to check that there was no failure of the modularity optimization algorithm to resolve small modules. Community/modular structure was reported for a resolution parameter of one which indicates the optimization algorithm was adequate for the size of the networks in the current study. Resolution difficulties are more problematic for very large networks and given the relatively small size of our networks (based on the 148 nodes of Destrieux parcelation) the magnitude of the resolution parameter was as expected (Fortunato and Hric, 2016).

### Sparse estimation

The Lasso sparse estimation technique required a regularization parameter (ρ). The regularization parameter was calculated using cross-validation based on the data for all participants, ρ = 0.184.

### Network parameter differences for the whole group

For the whole group, local efficiency increased from 0.501 at Time 1 to 0.511 at Time 2; global efficiency changed from 0.534 to 0.535; and modularity increased from 0.712 to 0.731. It was not possible to use a comparative analysis approach (non-parametric permutation tests) for the whole group topological properties because there was no comparison group.

### Network parameter differences between parenting groups

Comparative analyses (non-parametric permutation tests) of network parameters (local efficiency, global efficiency, and modularity) were performed for each of the four parenting components (Negativity EPI, Warmth, Negativity PSI, and Communication) at Time 2 and between the two time points (Time 1 and Time 2).

The *cross-sectional* comparisons for local efficiency, global efficiency and modularity at Time 1 were previously reported for the larger sample (*N* = 145; Richmond et al., 2019, 2021). Note that these results were based on three parenting groups (low-, moderate- and high-, see “Family interaction assessment and measures”). The Time 1 results presented in Tables 3–5 are based on the smaller sample (*n* = 114), for two parenting groups (low- and high-) and have not been compared cross-sectionally using non-parametric permutation testing.

**TABLE 3:** Mean local efficiency and group differences for low- and high-parenting components, time 1 and 2 (*n* = 114).

Parenting component	Low-	High-	Low–High (*p*)
Negativity EPI			
Time 1	0.452	0.443	0.009^a^
Time 2	0.465	0.463	0.001 (0.937)
Time 2–Time 1	0.013	0.020	
Omnibus *p*	0.415		
Warmth			
Time 1	0.450	0.468	−0.018^a^
Time 2	0.456	0.484	−0.029 (0.102)
Time 2–Time 1	0.005	0.016	
Omnibus *p*	0.527		
Negativity PSI			
Time 1	0.461	0.451	0.010^a^
Time 2	0.449	0.457	−0.008 (0.657)
Time 2–Time 1	−0.012	0.005	
Omnibus *p*	0.623		
Communication			
Time 1	0.451	0.479	−0.028^a^
Time 2	0.451	0.461	−0.010 (0.567)
Time 2–Time 1	0.013	0.020	
Omnibus *p*	*0.471*		

EPI, event-planning interaction; PSI, problem-solving interaction. ^a^Non-parametric permutation testing not conducted for Time 1 cross-sectional results. FDR (5%) adjusted *p-values*.

**TABLE 4:** Global efficiency and group differences for low- and high-parenting components, time 1 and 2 (*n* = 114).

Parenting component	Low-	High-	Low–High (*p*)
Negativity EPI			
Time 1	0.540	0.542	−0.002^a^
Time 2	0.538	0.547	−0.008 (0.260)
Time 2–Time 1	−0.002	0.004	
Omnibus *p*	0.996		
Warmth			
Time 1	0.548	0.542	0.006^a^
Time 2	0.541	0.548	−0.007 (0.313)
Time 2–Time 1	−0.007	0.007	
Omnibus *p*	0.966		
Negativity PSI			
Time 1	0.546	0.543	0.003^a^
Time 2	0.546	0.534	0.13 (0.077)
Time 2–Time 1	0.001	−0.009	
Omnibus *p*	0.916		
Communication			
Time 1	0.541	0.542	−0.001^a^
Time 2	0.534	0.540	−0.006 (0.418)
Time 2–Time 1	−0.002	0.004	
Omnibus *p*	0.977		

EPI, event-planning interaction; PSI, problem -solving interaction. ^a^Non-parametric permutation testing not conducted for Time 1 cross-sectional results. FDR (5%) adjusted *p*-values.

**TABLE 5:** Modularity (participation coefficient) and group differences for low- and high-parenting components, time 1 and 2 (*n* = 114).

Parenting component	Low-	High-	Low–High (*p*)
Negativity EPI			
Time 1	0.710	0.688	−0.022^a^
Time 2	0.702	0.699	0.003 (0.909)
Time 2–Time 1	0.007	0.012	
Omnibus *p*	0.729		
Warmth			
Time 1	0.741	0.713	−0.028^a^
Time 2	0.689	0.750	−0.061 (0.015*; 0.05 corrected)
Time 2–Time 1	0.053	0.037	
Omnibus *p*	0.119		
Negativity PSI			
Time 1	0.696	0.633	−0.063^a^
Time 2	0.671	0.694	−0.023 (0.370)
Time 2–Time 1	0.025	0.061	
Omnibus *p*	0.071		
Communication			
Time 1	0.699	0.703	−0.004^a^
Time 2	0.645	0.691	−0.045; *p* = 0.073
Time 2–Time 1	0.054	0.013	
Omnibus *p*	0.108		

EPI, event-planning interaction; PSI, problem-solving interaction. ^a^Non-parametric permutation testing not conducted for Time 1 cross-sectional results. FDR (5%) adjusted *p*-values *p < 0.05.

#### Parenting and mean local efficiency

In the larger sample at Time 1, higher levels of observed negative affective and communicative maternal behaviors were associated with decreased local network efficiency. In contrast, higher levels of positive affective maternal behaviors were associated with increased local network efficiency (Richmond et al., 2019).

At Time 2 (cross-sectional comparison), there were no significant group differences for any of the parenting components for mean local network efficiency (e.g., Low Warmth Time 2–High Warmth Time 2; Table 3). There were also no significant group differences for change in local network efficiency from Time 1 to Time 2 (Table 3).

#### Parenting and global efficiency

In the larger sample at Time 1, no support was found for an association between global efficiency and maternal behaviors (Richmond et al., 2019).

At Time 2 (cross-sectional comparison), there were no significant group differences for any of the parenting components for global network efficiency (Table 4). There were also no significant group differences for change in global efficiency from Time 1 to Time 2 (Table 4).

#### Parenting and modularity

In the larger sample, at Time 1, higher levels of negative maternal behavior were associated with lower modularity. No support was found for an association between positive maternal behaviors and modularity and between maternal communicative behaviors and modularity (Richmond et al., 2021).

At Time 2, For maternal Warmth, there was a significant difference in the participation coefficient where High-Warmth was associated with a higher participation coefficient than Low-Warmth (Table 5). For all the remaining parenting components, there were no significant group differences for the participation coefficient at Time 2 (Table 5). There were also no significant group differences for change in participation coefficients from Time 1 to Time 2 (Table 5).

## Discussion

This study demonstrates associations between maternal affective parenting behaviors (measured when children were 8 years) and the segregation of SC networks across late childhood. We hypothesized that more negative and less positive parenting behaviors would be associated with lower local efficiency and modularity at age 10 and, based on our prior findings, larger changes in local efficiency and modularity from age 8 to 10, which may reflect accelerated development. Associations were found, however, they were not all as predicted. No support was found for a relationship between maternal affective parenting (positive and negative) and local efficiency at age 10. Less positive affective maternal parenting was associated with higher modularity at age 10, whereas negative parenting was not related to modularity. No support was demonstrated for associations between maternal affective parenting behaviors and change in network efficiency and modularity, from age 8 to 10. As predicted, affective parenting behaviors were not associated with global efficiency at age 10.

Overall, the lack of support for a relationship between affective maternal parenting and local efficiency at age 10 was not expected. When the children were younger (age 8), for non-optimal parenting behaviors (i.e., high negative and low positive) we found a cross-sectional negative association with lower mean local efficiency (Richmond et al., 2019). Of importance, maternal behaviors were only measured at the first time point and therefore it is possible that these behaviors were not consistent across the (approximate) 2-year timeframe of the study and this may explain the lack of findings. This explanation, however, is contradicted by the association between less positive affective maternal behaviors and higher modularity (i.e., more segregation) at age 10. If we consider high negative and low positive to be “non-optimal” parenting, this is different to the pattern found previously at age 8, where higher negative maternal behaviors were associated with lower modularity (less segregation; Richmond et al., 2021).

The results indicate that while high levels of negative affective maternal behavior and low levels of positive affective maternal behavior can both be considered “non-optimal,” they may have differential effects. That is, an absence of maternal warmth (i.e., positive behavior) may have a different impact on children’s brains compared to high levels of maternal aggression and conflict (i.e., negative behavior). Alternatively, the impact of these behaviors may be different at different ages; that is, there may be a temporal specificity of associations between maternal behaviors and children’s structural brain networks. Emerging knowledge of the developmental trajectory for modularity suggests that modularity reaches a low in late childhood and then increases into adolescence (Khundrakpam et al., 2013; Nie et al., 2013). This is consistent with the pattern of modularity observed for the whole group where modularity increased from 8 to 10 years. Our results may thus imply that children exposed to low maternal warmth may be ahead on the trajectory and hence modularity is increasing compared to those exposed to high maternal warmth. The developmental timing of other environmental factors has been associated with children’s brain structure, for example, in the association between income to needs ratio and hippocampal and amygdala volumes (Ramphal et al., 2021) and indeed for parenting and CT from early to middle childhood (Chad-Friedman et al., 2021). Further work is needed to map the development of key network properties across development. This work could be expanded to relate brain changes to behavior and mental health to establish whether these changes are adaptive or maladaptive.

There were no associations between maternal affective behaviors and the magnitude of the change in network efficiency (local and global) or modularity, from 8 to 10 years, which was not as predicted. We expected that the associations that have been observed cross-sectionally would also translate to longitudinal change. To illustrate using modularity, as discussed, the results tentatively indicate that less positive maternal behavior (measured at age 8) is associated with an accelerated position along the typical developmental trajectory at age 10. We anticipated that children who experience less positive maternal behavior would also have a larger rate of change, however, this was not reflected in the results. This finding may suggest that maternal affective behaviors have an impact on network properties in early development, that is children are set off on a different developmental trajectory, but the rate of change along the trajectory is constant. If this was a plausible explanation then we would also have expected to see the association between less positive maternal behavior and modularity at Time 1, which was not supported (Richmond et al., 2019). The results indicate maternal warmth (measured at age 8) was only related to modularity when the children were older. Again, as mentioned previously, there is a clear need to map the impact of environmental factors and the development of key network properties across development.

As predicted, global efficiency was not associated with affective parenting behaviors at age 10. This was consistent with our previous finding at age 8 (Richmond et al., 2019). The pattern observed for the whole group indicated that global efficiency remained constant from 8 to 10 years. Consistent with the notion of neural “plasticity” and sensitive periods (Gee, 2022), it may be that lack of change in global efficiency over the developmental period of the current study, means that this network property is less amenable to change as a result of maternal affective behavior. Our finding, however, is not consistent with Khundrakpam et al. (2013) who found late childhood to be a time of increased global efficiency. However, comparing findings across studies of SC is problematic, because there are typically differences in analytical approaches ranging from parcelation schemes to generating sparse matrices.

The results of the current study further our understanding of the relationship between parenting and brain network development but are not without limitations. We focused on maternal behaviors and did not consider the bi-directional nature of the dyadic interactions between children and their mothers and therefore cannot assess the role of the child’s behavior in the interactions. Further, our primary aim was to investigate parenting behaviors and structural connectivity and we did not investigate associations between parenting behaviors and CT. Our group, incorporating the same sample as the current study, has demonstrated that parenting behaviors predict other aspects of brain function and structure. Positive parenting has been linked to resting state functional connectivity of higher order control networks (Pozzi et al., 2021) and harsh and inconsistent parenting with altered cortical development (Whittle et al., 2022). Taken together, the evidence indicates that different neural metrics reveal differences in the impact of parenting on the developing brain.

In addition, as mentioned above, SC is a relatively new method for constructing brain networks and the existing literature base includes different analytical approaches. Previous work has typically considered the influence of covariates (i.e., multiple scanners, age, etc.) through the use of linear regression with correlations based on extracted residuals (Teicher et al., 2014; Khundrakpam et al., 2017). In contrast, in the current study, there were no group differences between potentially confounding variables, and we used *partial* correlations to generate networks (i.e., correlations of CT after removing variance shared with other nodes; He et al., 2008; Teicher et al., 2014). Further, as age was tightly controlled and all neuroimaging data were acquired on the same scanner, neither of these variables warranted attention. A related limitation was our approach to the parenting components. We divided participants into two equal groups based on median split to maintain a minimum group size for the sparse estimation technique. The groupings do not necessarily represent theoretical cut-off points for “high” and “low” levels of the parenting components and therefore the results should be interpreted with caution. One of the strengths of the current study is that the SC methodology applied is consistent with the previous cross-sectional approach (Richmond et al., 2019, 2021) which enables comparisons. The results, however, should be interpreted with caution.

This paper is the first to explore associations between normative variations in maternal parenting behaviors and structural brain networks longitudinally. The results provide preliminary evidence that variations in the emotional climate of typical family environments may be associated with the modularity of SC networks in children. As the results demonstrate a link between only one of the network measures (modularity) and one of the parenting behaviors (Warmth) for children aged 10 years but not 8 years, they should be interpreted with caution. It would be of interest for future work to clarify whether these brain network differences are related to the link between parenting and the development of mental health difficulties in childhood (Yap and Jorm, 2015).

## Data availability statement

The raw data supporting the conclusions of this article will be made available by the authors, without undue reservation.

## Ethics statement

The studies involving human participants were reviewed and approved by the human research Ethics Committee at The University of Melbourne [Office of Research Ethics and Integrity (OREI)]. Written informed consent to participate in this study was provided by the participants’ legal guardian/next of kin.

## Author contributions

SW, MS, and NA contributed to conception and design of the broader FACTS study and obtained funding. SR, KB, and EP were involved in data collection and image processing for FACTS. SW, MS, NA, RB, and KJ provided guidance and feedback on the design of the current study. SR performed the statistical analysis with consultation provided by RB and wrote the first draft of the manuscript. All authors contributed to manuscript, read, and approved the submitted version.
